# Phase Behavior and Thermo-Mechanical Properties of IF-WS_2_ Reinforced PP–PET Blend-Based Nanocomposites

**DOI:** 10.3390/polym12102342

**Published:** 2020-10-13

**Authors:** Ding Chen, Santosh K. Tiwari, Zhiyuan Ma, Jiahao Wen, Song Liu, Jiewei Li, Feng Wei, Kunyapat Thummavichai, Zhuxian Yang, Yanqiu Zhu, Nannan Wang

**Affiliations:** 1 College of Chemistry and Chemical Engineering, Guangxi University, Nanning 530004, China; 1914402004@st.gxu.edu.cn (D.C.); 1610201024@st.gxu.edu.cn (J.L.); 2 Key Laboratory of New Processing Technology for Nonferrous Metals and Materials, Ministry of Education, Guangxi Institute Fullerene Technology (GIFT), School of Resources, Environment and Materials, Guangxi University, Nanning 530004, China; ismgraphene@gmail.com (S.K.T.); 1714402023@st.gxu.edu.cn (Z.M.); 1814202016@st.gxu.edu.cn (J.W.); 1710201026@st.gxu.edu.cn (S.L.); 1610201014@st.gxu.edu.cn (F.W.); 3 College of Engineering, Mathematics and Physical Sciences, University of Exeter, Exeter EX4 4QF, UK; kt302@exeter.ac.uk (K.T.); Z.Yang@exeter.ac.uk (Z.Y.)

**Keywords:** IF-WS_2_, PP/PET blend, nanomaterials, phase behavior, thermal-mechanical properties

## Abstract

The industrial advancement of high-performance technologies directly depends on the thermo-mechanical properties of materials. Here we give an account of a facile approach for the bulk production of a polyethylene terephthalate (PET)/polypropylene (PP)-based nanocomposite blend with Inorganic Fullerene Tungsten Sulfide (IF-WS_2_) nanofiller using a single extruder. Nanofiller IF-WS_2_ was produced by the rotary chemical vapor deposition (RCVD) method. Subsequently, IF-WS_2_ nanoparticles were dispersed in PET and PP in different loadings to access impact and their dispersion behavior in polymer matrices. As-prepared blend nanocomposites were characterized by scanning electron microscopy (SEM), transmission electron microscopy (TEM), thermogravimetric analysis (TGA), dynamic differential scanning (DSC), dynamic mechanical analysis (DMA), and X-ray diffraction (XRD). In this work, the tensile strength of the PP/PET matrix with 1% IF-WS_2_ increased by 31.8%, and the thermal stability of the sample PP/PET matrix with 2% increased by 18 °C. There was an extraordinary decrease in weight loss at elevated temperature for the nanocomposites in TGA analysis, which confirms the role of IF-WS_2_ on thermal stability versus plain nanocomposites. In addition, this method can also be used for the large-scale production of such materials used in high-temperature environments.

## 1. Introduction

Novel developed materials are playing key role in our day-to-day life and it is problematic to imagine the present world without polymers and their derivatives. Owing to tunable properties, such as chemical inertness, thermo-mechanical stability along with low density, high elasticity, good transparency, suitable toughness, and relaxed processing it is an ideal material for cutting-edge technologies [1,2]. Among the various classes of polymeric materials, polymer blends are performing important roles from entertainment to medical care, from clothing to the food industry, and from communication to the defense industries [3].

Polyethylene terephthalate (PET) and polypropylene (PP) are widely used materials for engineering applications and nearly 65 and 35 million tons are produced annually, respectively [4]. PET is a polar, aromatic polymer, whereas PP is non-polar, semi-crystalline material with low thermal stability [5]. Several blends have been developed using PP with other polymers including PET for different purposes, and many of them are part of important industries [6]. Recently, PP has been blended with some high-melting-point polymers such as PET, Nylon, Ethylene Vinyl Acetate Copolymer (EVA), etc. using different kinds of nanofillers as discussed elsewhere to increase miscibility and to minimize phase separation [7,8]. The main nanofillers presently used to reinforce the properties of blend nanocomposites are graphene, graphene oxide, and single/few-layer Boron Nitride (BN) [9,10]. The aforementioned nanofillers have their own advantages and disadvantages, but have great impact on new material innovations.

Presently, 5G communications, polymer-based chips, and electronic components are in high demand and require a lot of innovation. Thus, improving the thermal properties of materials such as PP has become a big opportunity for electronic communication and next-generation computer technologies [11,12]. Similarly, the development of heat-removal materials with good thermal properties and low cost could also be undertaken using such polymeric materials [13]. Owing to the characteristics of PP, it is a primary structural material for 5G antennas, but it needs several modifications for effective and durable service [14]. Considering this, several reports that blend PP with PET could be the best choice to achieve excellent toughness, wear resistance, chemical stability, and thermal stability [15,16]. However, PET as a reinforcing phase with PP to make copolymer materials may be a convenient method to improve thermal stability, because a neat PP–PET blend usually exhibits rough, unstable phase morphology, and poor mechanical properties [17]. Moreover, weak adhesion at interfaces in the case of neat blends results in cracking, especially with time. Such detracting behavior of neat blends directly depends on the viscoelastic nature of blending partners as well as on the orientation of the one component over the others during mixing, and cannot be controlled without a suitable filler [18]. For this particular application, a carbon-based nanofiller will not be a good choice for several reasons, and we need suitable inorganic fullerene-like (IF) nano-systems.

During last three decades, inorganic fullerene-like nanostructures and their analog-based transition metal dichalcogenides have attracted much research interest, and polymer technologies are similar [19,20,21,22]. To date, numerous IF-MS_2_ (M ¼ W, Mo, Re, Tb, etc.) nanomaterials have been synthesized and show amazing lubricating, mechanical, and electronic properties [23,24,25]. The incorporation of these nanomaterials into polymer matrices such as PP, PC, LDPE, and Nylon were investigated in the past few years and greatly improved thermo-mechanical properties were realized, which are presented in Table 1. Among others, IF-WS_2_ shows amazing thermal stability along with astonishing shock-absorbing performance [26]. In addition, bulk production of IF-WS_2_ is well optimized (fabricated by rotated chemical vapor deposition method) and its physical properties, such as size, thickness, and thermal stability, can be easily tuned. Thus, WS_2_ may be a potential candidate to fabricate lightweight and high-performance polymer-blend nanocomposites for industrial applications [27].

Overall, this work aims to reinforce the compatibility and thermal stability of a PP/PET blend by mixing with the hollow structure of IF-WS_2_ nanoparticles. For this purpose, PET is the reinforcing polymer with an addition of 20%, and PP is the matrix with adjoining of 80%. A melt extrusion method was performed for the dispersion of different mass fractions of IF-WS_2_ nanoparticles into the PP/PET blends. The ultimate performance of blends was investigated on thermal and mechanical properties. Meanwhile, the enhancement of these properties will enable this new type of nanomaterial to be widely developed into many crucial areas in industry.

## 2. Experiment Section

### 2.1. Materials

Polypropylene (PP, model specification, M2500E) with MFI = 2.5 g/10 min at 200 °C/5 Kg was purchased in this study from Shanghai Petrochemical Co. Ltd. (Shanghai, China), as white transparent. PET (model specification, Dupont 530) was supplied from E. I. DuPont de Nemours and Company, Wilmington, DE, USA. The IF-WS_2_ nanoparticles were synthesized by Xu Fang’s rotary furnace reaction method [19]. The average size is 90 nm (80–110 nm).

### 2.2. Preparation of IF-WS_2_/PP/PET Nanomaterials

**Figure 1:**
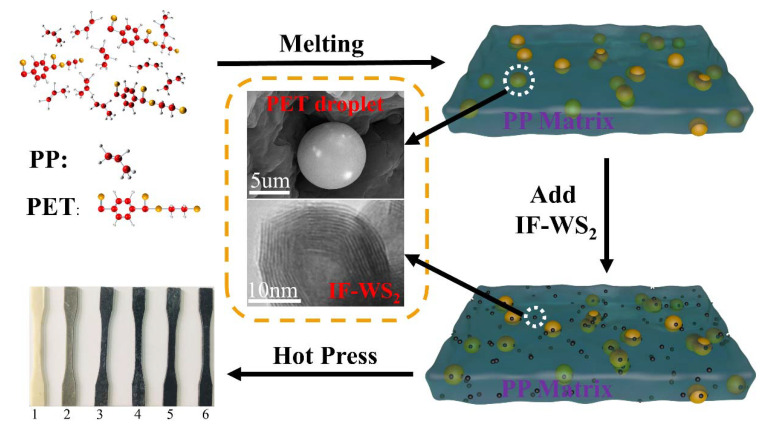
The schematic of the synthesis of nanomaterials with different fracture of IF-WS_2_.

The nanocomposites and PP/PET blends were prepared in a single screw extruder (KelesSJ-30, Jiangsu, China, Temperature of each four zones 200 °C, 210 °C, 210 °C, and 215 °C, with a screw speed 50 r/min). The schematic of the synthesis is shown in Figure 1.

Before blend-melt mixing, IF-WS_2_ nanoparticles were dried for 2 h at 80 °C, PP was dried for 4 h at 70 °C, and PET was dried 4 h at 120 °C. The specimens were fabricated by hot-pressing machine (Yuanfeng-8017, Yangzhou, Jiangsu, China) at 230 °C, 16 MPa. The composition of different nanocomposites and blends in this study is shown in Table 2.

### 2.3. Characterization of IF-WS_2_/PP/PET Nanocomposites

Scanning Electron Microscopy (SEM, Hitachi SU8220, Chiyoda, Tokyo, Japan) was used for observing the morphology of IF-WS_2_/PP/PET nanocomposites, 15 kV operational voltage, and the samples were pre-coated with a thin gold layer. X-ray diffraction (XRD, Rigaku D/MAX 2500V, Matsubara-cho, Akishima-shi, Tokyo, Japan) was operated to appraise the crystal structure analysis, a step size of 0.03°, over a 2θ range of 10–80°. Transmission electron microscopy (TEM, FEI TECNAI G2 F30, Hillsboro, OR, USA) was performed to study the micrographs of the IF-WS_2_ nanoparticles and the actual particle distribution of the matrix. Also, the test nanocomposite sample was prepared into 0.3 × 0.3 mm by a diamond razor blade, with a sample thickness of 100 nm during the TEM test. The mechanical properties of nanocomposites (Dumbbell shape) were tested by material mechanics experimental system (British model specification 8801, INSTRON, Norwood, MA, USA), with test speed of 50 mm/min at room temperature and the specimen. At least 5 samples were tested for each concentrate of materials. Thermogravimetric analysis (TGA, Perkin Elmer-4000, Waltham, MA, USA) of neat blends and nanocomposites were carried out under a dried nitrogen atmosphere at a scan rate 10 °C/min in the temperature range 30–800 °C. The melting temperature and crystallization temperature curves of the nanocomposite were obtained from dynamic differential scanning (DSC, model specification, TA instruments-Model 25, New Castle, DE, USA). Dynamic mechanical analysis (DMA, TA instruments-Model 850, New Castle, DE, USA) was characterized in the tensile mode at a frequency of 1 Hz, with rectangular geometry films, at temperatures from 30–200 °C. Specimens of the storage modulus (E’) and as a function of the temperature were appraised using a heating rate of 3 °C/min.

## 3. Results and Discussion

### 3.1. Structure and Morphology Analysis of IF-WS_2_ Nanoparticles

**Figure 2:**
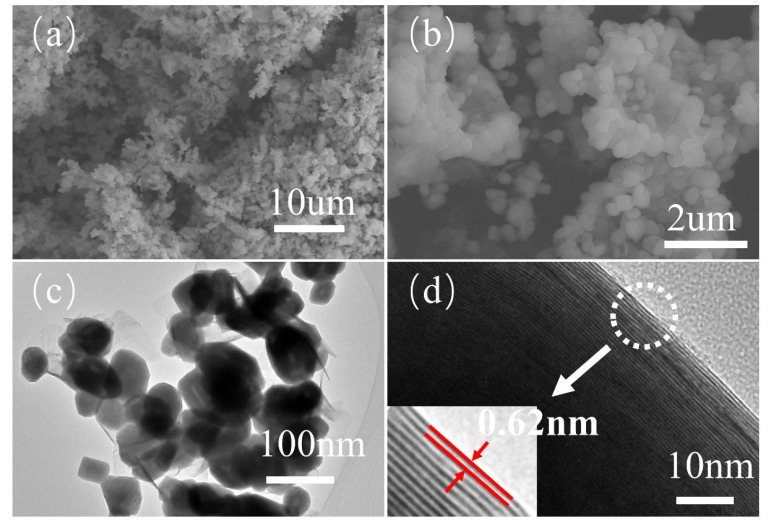
SEM (**a**,**b**) and TEM (**c**,**d**) images of IF-WS_2_ nanoparticles.

The morphology and structure of IF-WS_2_ nanoparticles were characterized by SEM and TEM in Figure 2. As shown in Figure 2a,b, the IF-WS_2_ nanoparticles presented spherical and hemispherical nanostructures with a size of about 90–110 nm. The hollow-cage configuration of nanoparticles is clearly shown in Figure 2c,d. Meanwhile, it can be noticed that there are multiple closed-ring structures at the edges of the nanoparticles in Figure 2d.

### 3.2. SEM Analysis

**Figure 3:**
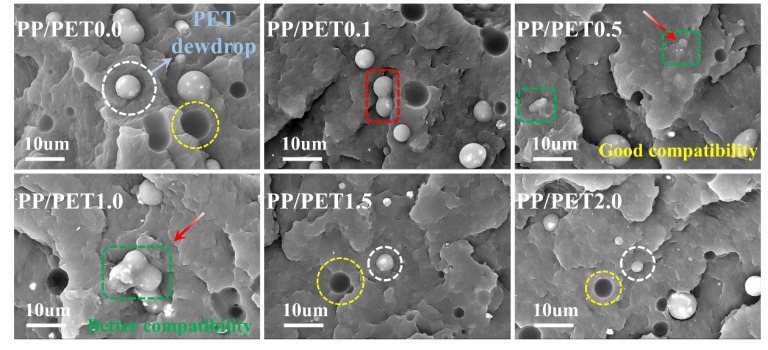
Scanning electron microscopy (SEM) microstructure graphs with backscattered electron image (BSE) mode of cryogenically fractured surface under liquid nitrogen with different mass fraction of IF-WS_2_:PP/PET0.0, neat blend; PP/PET0.1, 0.1 wt% IF-WS_2_; PP/PET0.5, 0.5 wt% IF-WS_2_; PP/PET1.0, 1 wt% IF-WS_2_; PP/PET1.5, 1.5 wt% IF-WS_2_; PP/PET2.0, 2 wt% IF-WS_2_.

The SEM images of the fractured surface of neat blend and nanocomposites are presented in Figure 3. These images demonstrate an imaginable mechanism for reinforcement owing to the incorporation of IF-WS_2_ as a nanofiller in nanocomposites. The pure PP–PET blend with clear droplet and cracked structure is signified, which is owing to the immiscibility controlled by the viscosity hysteresis between PP and PET. Furthermore, it is believed that the droplet is a form of PET due to the high shear pressure extracted from PP during the melting and compounding process. The droplets can be seen clearly. Meanwhile, the cracks and open-ring structures (as indicated by the circle) can be seen more clearly in the magnified SEM image of the pure PP–PET blend. Indeed, a very highly incompatible property of PP and PET blend was confirmed by SEM observation reported elsewhere [17]. Moreover, with the addition of IF-WS_2_ into PP–PET by 0.1%, an indistinctive change in surface morphologies of nanocomposite was exhibited in Figure 3 (PP/PET0.1). In addition, little decrease in the size of PP phase along with the collapsing of droplets (coalescence phenomenon) into the surface can be observed in the same picture. The exact reason for coalescence of the polymer blends in the presence of IF-WS_2_ has been elaborated on elsewhere [18]. However, even in this case, cracking, droplets, and phase separation is quite observable and similar to the pure PP–PET system. Upon further loading, the same surface morphology was observed in the blend with 0.1% of IF-WS_2_. The degree of compatibility seems much better in the case of 0.5% loading owing to the nanosizing effect, decrease in droplet size, and increased miscibility (Figure 3 PP/PET0.5). Interestingly, 1% of IF-WS2 (Figure 3 PP/PET1.0) was loaded into the PP–PET blend, showing a high degree of compatibility, no cracks at the interface, and huge coalescence. In this case, the SEM image of blend nanocomposite shows no individual phases of PP and PET, which is an unswerving sign for sufficient loading of nanofiller to the blend system. In this case, the separate phases of PP and PET were not displayed. This is an unwavering sign that the nanofiller is sufficient to be loaded into the blended system. Moreover, the roughness of the surface in the case of 1% of IF loading suggests better adhesion at the PET–PP interface and better thermo-mechanical properties (Figure 3 PP/PET1.0). Therefore, the 1% IF-WS_2_ loading showed a complete change in the obvious aggregation morphology of the PP–PET phase, which may be due to the nucleation of IF nanoparticles in PET and promote the collapse of the droplets. Surprisingly, droplet formation, cracking, and diminished coalescence phenomenon are described with the further increase of IF-WS_2_ loading (such as 1.5% and 2% loading) in Figure 3 (PP/PET1.5, PP/PET2.0). This is mainly due to the aggregation of nanofillers in the blend matrix, which is related to the decrease in the mechanical properties of blend PP/PET1.5 and PPPET2.0 [28].

### 3.3. TEM Analysis

**Figure 4:**
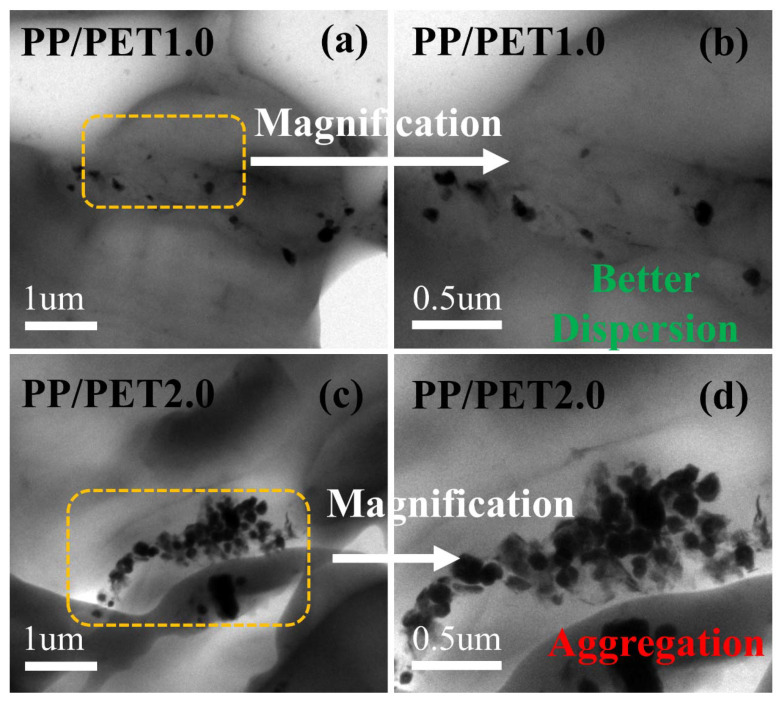
Transmission electron microscope (TEM) graphs of the PP/PET blend-based nanocomposites with 1 wt% (**a**,**b**) and 2 wt% (**c**,**d**) IF-WS_2_ nanoparticles at different magnification.

The exact distribution of IF-WS_2_ in PP–PET was auxiliary deliberated using the high resolution transmission electron microscope (HRTEM) technique. Here we are only presenting TEM images of polymer blends with 1% and 2% of IF-WS_2_ instead of all samples. The logic behind selecting only these two samples is similar to the SEM morphology remaining samples. Figure 4 (PP/PET1.0) shows typical TEM pictures of the PP–PET sample containing 1% IF-WS_2_, and nanofillers can be seen at the interfaces of PP and PET. Moreover, IF-WS_2_ is well dispersed in both phases, and IF-WS_2_ nanoparticles can be seen in microtome slice sample. The light gray and dark gray parts in the TEM image correspond to PP and PET blends, respectively. The uniform dispersion of IF-WS_2_ may be due to the positive interaction between the oxygen functional groups of the nanofiller and the polar groups of PET. If pragmatic, all IF-WS_2_ were mostly localized in the PET polymer due to its viscoelastic property along with significant amount rapped on PP polymer chains. The TEM images of blend consisting of 2% of IF-WS_2_ and highly aggregated IF-WS_2_ can be seen in PET along with a few portions of interfaces as well (Figure 4, PP/PET2.0).

### 3.4. Tensile Properties

**Figure 5:**
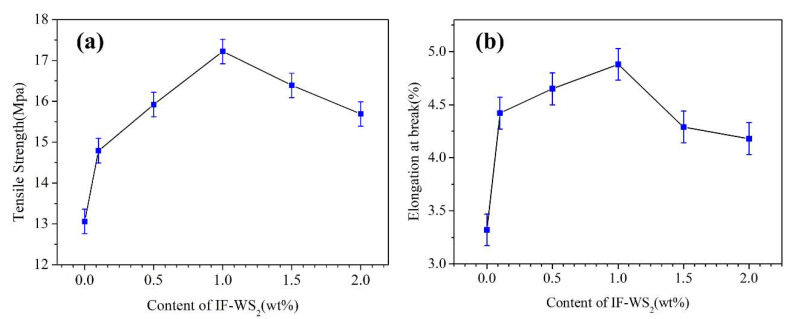
(**a**) Average tensile strength (MPa) values, (**b**) elongation at break (%) rates of the blend/IF-WS_2_ nanocomposites as a function of IF-WS_2_ contents.

The tensile strength and elongation at break (%) of pure blend and nanocomposites with different amounts of IF-WS_2_ (0.1 wt% to 2 wt%) are shown in Figure 5. Typical tensile strength curves of the neat blend and nanocomposites clearly demonstrate a distinct improvement in tensile strength on the appropriate addition of IF-WS_2_ owing to the nucleation and nanosizing effect. The value of tensile strength of samples very clearly implies a role of different loading of IF-WS_2_, and as a result was against the plain blend sample. The 1 wt% composite presented a 31.8% improvement in the ultimate tensile strength, which increased from 13.06 MPa to 17.22 MPa in Table 3. This can be attributed to the bridging effect of IF-WS_2_ which functions as a stress-transferring medium between the blending partners and agrees with previous studies [24,26]. Here, outcomes show the nanocomposite with 1% of IF-WS_2_ had the highest value of tensile modulus compared to all other samples, and a further increase in loading to 2% shows a declined trend in the tensile strength, which may be due to agglomeration (due to excess loading) of IF-WS_2_ in the blend matrix and hence the appropriate loading is crucial to enhancing thermo-mechanical properties. It mostly happened due to preferential dispersion of IF-WS_2_ in the minor PET phase and controlled by polar–polar interaction at optimum loading. The role of different loading of IF-WS_2_ is also discussed in detail in the TGA and DSC section.

### 3.5. XRD Analysis

**Figure 6:**
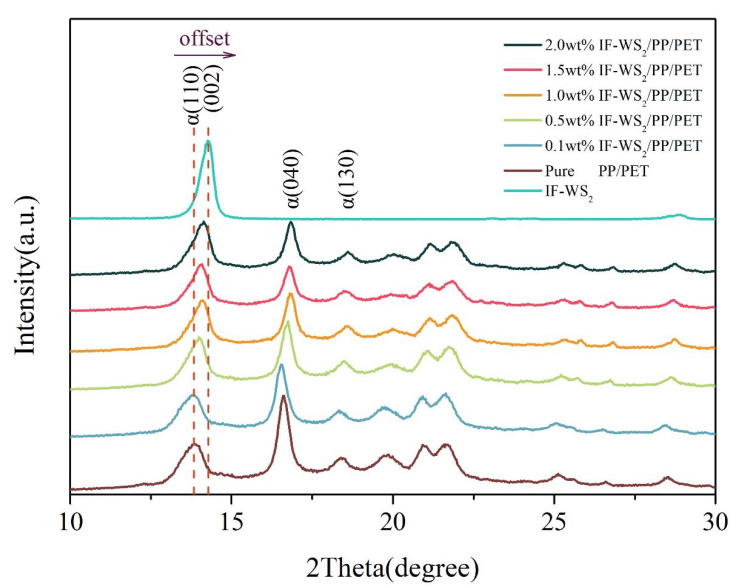
X-ray diffraction (XRD) patterns of IF-WS_2_ nanoparticles and blend/IF-WS_2_ nanocomposites with different amounts of IF-WS_2_ contents.

The impact of different loadings of IF-WS_2_ on crystallinity of prepared polymer nanocomposites was investigated using XRD and plots are presented in Figure 6. The effect of different loadings of IF-WS_2_ on the crystallinity of the fabricated nanocomposites was observed using XRD technology. The semi-crystalline property of the pure blend and nanocomposite materials was different due to the shift of the IF-WS_2_ loading in Figure 6. In the XRD plot, the characteristic diffraction peak for the PP and PET agree well with previous results [39]. However, a slight change in the peak position (especially in change in width) in the case of the blends containing IF-WS_2_ can be perceived when equated to the pure blend. As a semi-crystalline plastic, PP matrix has characteristic diffraction peaks of 2θ = 14.1°, 17.1° and 18.6°, corresponding to the planes (110), (040), and (130), which are typical reflections of α-crystals. In addition, it can be seen that the characteristic diffraction peak of the pure copolymer matrix with the content of IF-WS_2_ has a significant shift to the (002) characteristic diffraction peak of IF-WS_2_. The analysis suggests that this may be due to the interaction of the peak of IF-WS_2_ and the blend. Interestingly, most of the well-defined peak positions in the figure for as-synthesized nanocomposites are closer to peaks for PET and PP, either merged or very tiny. The blends indicate a semi-crystalline property, and a new bond structure is clearly expected from the observations of different peak positions of the newly fabricated materials. The XRD peaks for 0.1% and 0.5% IF-WS_2_ loading are very similar to each other and it is very difficult to see any measure of impact of the nanofiller. This result is consistent with SEM and tensile results. On the other hand, in the case of 1.5 and 2% IF-WS_2_ loading, sharpness in one of two prominent XRD peaks get pronounced, which may be attributed to the aggregation of IF-WS_2_ perennially in the PET phase, while PP is strapped outside and result low thermal stability as discussed in TGA/DSC section. Undeniably, both prominent peaks get equally sharp and intense in the case of 1% of IF-WS_2_ loading. It implies 1% IF-WS_2_ is sufficient and could be the best content for PP–PET-based polymer nanocomposites. Moreover, predominant deposition of IF-WS_2_ in only the PET phase can disrupt the non-bonding interactions among polymer chains acting as a barrier between two chains [40]. The excess dispersion of IF-WS_2_ in PET phase could be due to lower viscosity of PP as compared to PP at melting conditions.

### 3.6. TGA Analysis

**Figure 7:**
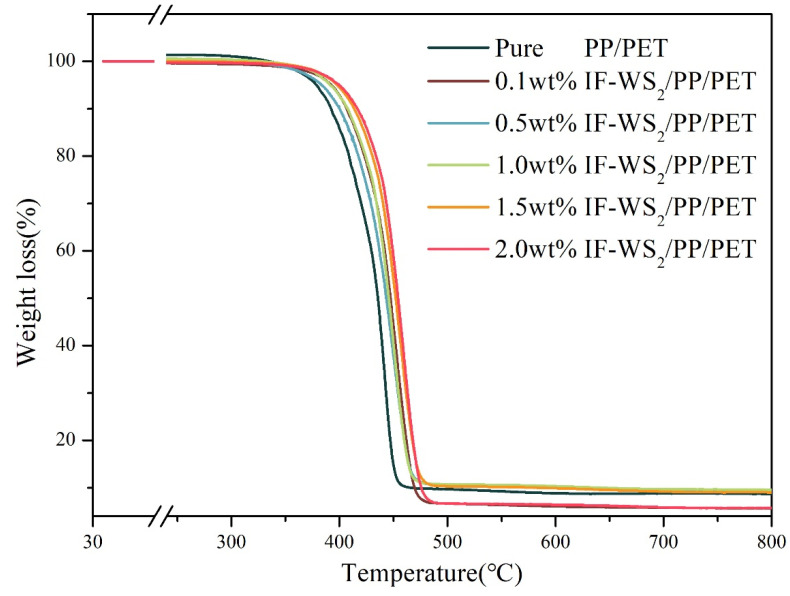
Thermogravimetric analysis (TGA) curves of PP/PET blends with different mass fraction of IF-WS_2_ nanoparticles.

To examine thermal properties and weight loss of neat blend and nanocomposites, TGA was conducted and a TG thermogram is presented in Figure 7. The data obtained from TGA is presented in Table 2. The data reveals that due to the incorporation of IF-WS_2_, T_s_ (onset degradation) was shifted toward a higher temperature. From Figure 7, it is also clear that the 1% IF-WS_2_ loading is enough for its homogeneous dispersion and greatly affects phase behavior of polymer nanocomposites, as discussed in the SEM section. It is well established that PP and PET start gigantic thermal decomposition above 280 °C and 250 °C, respectively, and its pure blend beyond 310 °C, as reported in [41,42]. However, in the present study, pure blend showed weight loss as expected, while weight loss of nanocomposites with IF-WS_2_ is significantly increased and can be observed in the graph. This reflection might be accredited to cross-liking between the PP and PET governed to the incorporation of IF-WS_2_ as discussed in the SEM and tensile properties sections. Thus, the nucleation and cross-liking nanosizing effect of IF-WS_2_ offers a combined response of both blending allies toward the uniform heat distribution during heating in TG testing. With the increase of IF-WS_2_ loading, the loading of IF-WS_2_ nanoparticles leads to enhanced crosslinking of the interface, delays the unfavorable enthalpy (increase of molecular motion) conditions, and leads to enhanced thermal stability. Under a load of 0.1% and 0.5%, the phase separation and crosslinking is weakened due to insufficient nanofillers. Although the thermal stability has increased under 1.5% and 2.0% loads, the enhancement was not obvious. Meanwhile, the char residue of different mass of IF-WS_2_/PP/PET nanocomposite can be clearly obtained in Table 4. In this study, the nanocomposite loaded with 1% IF-WS_2_ has the highest char residue, which is further confirmed when the nanocomposite has better thermal stability with 1% IF-WS_2_ loading. Therefore, the 1% load can make the nanocomposite have good thermal stability and economic efficiency.

### 3.7. DSC Analysis

**Figure 8:**
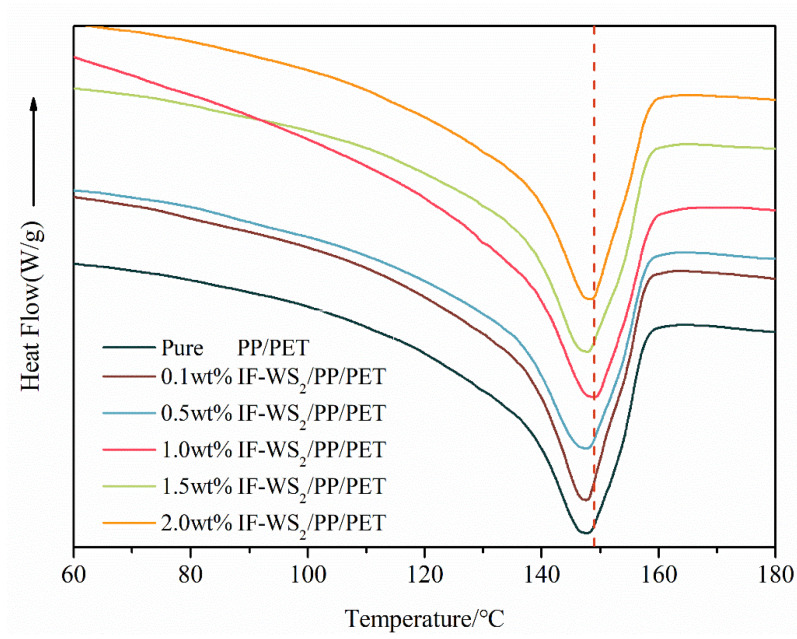
Dynamic differential scanning (DSC) second heating cycles of nanocomposites with different mass fraction of IF-WS_2_ nanoparticles.

The DSC curves of pure blend and nanocomposites is shown in Figure 8. the melting temperature of the nanocomposites is presented in Table 5. Since melting temperature of PP and glass transition temperature of PET comes near each other at around 80 °C, this region is analyzed for thermal characterization of fabricated systems. Similarly, merging of melting temperature and glass transition temperature of two different polymer has also been previously reported [7]. In the present case there is no peak in DSC thermogram instead of melting region 145–155 °C.

From the DSC graph, it is clear that the assimilation of IF-WS_2_ into PP–PET matrices made significant changes in thermal stability. The results shown that at a lower loading (0.1% and 0.5%) there is a diminutive increase in the melting point of PP, which recommends that the maximum nanofiller distributed in the PET is due to better interaction and its high viscosity at melting condition restrict migration of IF-WS_2_ as previously reported. On the other hand, higher loading (1.5% and 2%) decreased melting temperature, which is attributed to phase separation. Among the PP and PET, PET is quite polar while PP is non-polar. During melt-compounding, polar–polar interaction among PET and IF-WS_2_ might influence its dispersion in a PP–PET blending system. Such interaction of IF-WS_2_ first migrates to the PET phase and then to PP. However, in the case of higher loading, this migration is badly restricted by the higher viscosity of the PET phase, and results in the aggregation of nanofiller, as shown in the TEM section. Thus, the migration and uniform dispersion of IF-WS_2_ into polymer matrices is controlled by kinetic (viscosity lag between PP and PET) and thermodynamic effects (polar and non-polar interaction of PET with IF-WS_2_).

### 3.8. Storage Modulus Analysis

**Figure 9:**
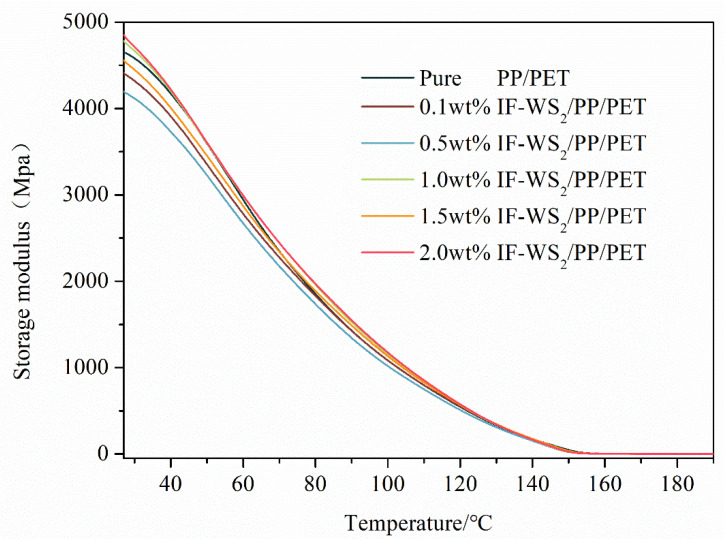
Dynamic mechanical behavior (storage modules) of blends and nanocomposites.

Variation in the storage modulus as a function of temperature for the as-fabricated polymeric systems is presented in Figure 9. A linear decrease in storage modulus with increase of temperature can be observed of up to 1% IF-WS_2_ loading and vice versa beyond the 1% of loading. This alteration can be attributed to the increase in molecular motion of the blend partners, owing to rapid thermal conduction and a resulting decrease in rigidity. This observation is in well in accordance with DSC results.

A notable increase in storage modulus for the PPPET1.0 in the elastomeric region due to the incorporation of IF-WS_2_ can be observed. The maximum increase in storage modulus for 1% can be attributed to the uninform dispersion of IF-WS_2_ in both PP and PET phases as discussed in the DSC and tensile testing sections. Also, a high aspect ratio of IF-WS_2_ increases the polymer–nanoparticle interaction area and hence the smooth stress transfers to enhance the storage modulus. However, increase in IF-WS_2_ loading to 2% expressions lowers the storage modulus as compared to PPPET1.0 blend nanocomposite, which can be attributed to the agglomeration of IF-WS_2_ as presented in the TEM section.

## 4. Conclusions

In summary, the uniform distribution of IF-WS_2_ in PP–PET composites has been efficaciously realized using melt-compounding methodology. Thermal stabilities and strengths of as-fabricated blend systems with increased loading of IF-WS_2_ were examined using DSC, TGA, DMA, and tensile instruments. This examination confirms improved compatibility between immiscible blending partners and facilitates enhanced stress transfer at the interface during thermo-mechanical testing. Thus, a strategy was extended to increase the compatibility of PP and PET wherein IF-WS_2_ localized at the interface enhanced the interaction between the blend components. Furthermore, selective dispersion of IF-WS_2_ and its effect on PP–PET morphological properties was observed for nanocomposites, signifying better stress transfer at the interface. A diagram exemplifying the formation of droplets on the surface of nanocomposites and the aggregation of IF-WS_2_ is also presented as a graphical abstract.

## Figures and Tables

**Table 1:** Summary of recent work about IF-WS_2_-based polymer nanomaterials to reinforce properties.

S.N.	Blending Partner	Focused Study	Ref.
1	PP + EVA	Thermal and dynamic mechanical properties	[28]
2	Nylon 12 (PA12)	Thermal stability and mechanical properties	[26]
3	Isotactic Polypropylene	Thermal behavior	[29]
4	Epoxy Adhesives	Toughness properties	[30]
5	Phenylenesulfide (PPS)	Isokinetic and iso-conversional study of dynamic crystallization kinetics	[31]
6	PP	Dynamic crystallization and melting behavior	[32]
7	PPS	The cold crystallization kinetics and mechanical performance	[33]
8	Poly ether ether ketone (PEEK)	Thermal and mechanical performance	[34]
9	PPS + PEEK	Rheological and Tribological Properties	[33]
10	PEEK	Thermal, mechanical, and tribological properties	[35]
11	ultra-high molecular weight polyethylene (UHMWPE)	Bulletproof properties	[36]
12	PEEK	Thermal, mechanical performance, and interface investigation	[25]
13	PPS + Isotactic polypropylene (iPP)	Mechanical and hardness properties	[37]
14	PVB	thermo-mechanical and tribological properties	[38]
15	Nylon 6 (PA6)	Dynamic crystallization and melting behavior	[26]

**Table 2:** Composition of different nanocomposites and blends investigated in this study.

Sample Code	PP (wt%)	PET (wt%)	IF-WS_2_ (wt%)
PP/PET0.0	80	20	0
PP/PET0.1	80	20	0.1
PP/PET0.5	80	20	0.5
PP/PET1.0	80	20	1.0
PP/PET1.5	80	20	1.5
PP/PET2.0	80	20	2.0

**Table 3:** Effect of IF-WS_2_ and nanocomposites on the Tensile strength (MPa) and elongation at break (%).

Sample Code	Tensile Strength (MPa)	Elongation at Break (%)
PP/PET0.0	13.06	3.32
PP/PET0.1	14.79	4.42
PP/PET0.5	15.92	4.65
PP/PET1.0	17.22	4.88
PP/PET1.5	16.39	4.29
PP/PET2.0	15.69	4.18

**Table 4:** Thermogravimetric analysis (TGA) critical points of IF-WS_2_/PP/PET nanocomposites under nitrogen flow at a heating rate of 10 °C/min (*T*_S_ represents the onset temperature of degradation, *T*_m_ represents the maximum temperature of decomposition, and *T*_50_ represents the decomposition temperature of the nanocomposite when the mass loss was 50%).

Sample Code	*T*_s_ (°C)	*T*_50_ (°C)	*T*_M_ (°C)	Char Residue at 500 °C (wt%)
PP/PET0.0	415	435	449	9.4%
PP/PET0.1	424	447	468	6.4%
PP/PET0.5	417	443	465	10.3%
PP/PET1.0	421	445	464	11.1%
PP/PET1.5	426	452	472	9.9%
PP/PET2.0	433	454	473	6.6%

**Table 5:** The melting temperature of nanocomposites with different fraction of IF-WS_2_.

Sample Code	Melting Temperature
PP/PET0.0	146
PP/PET0.1	146.6
PP/PET0.5	147.2
PP/PET1.0	148.9
PP/PET1.5	146.2
PP/PET2.0	147.5
